# Supplementary material for: The Systems Biology Research Tool: evolvable open-source software
Source: BMC Syst Biol. 2008 Jun 29;2:55. doi: 10.1186/1752-0509-2-55 (PMC2446383; doi:10.1186/1752-0509-2-55)
Supplement: Additional file 1 — SBRT Archive. An archive of the current version of the Systems Biology Research Tool. [file 1752-0509-2-55-S1.zip › sbrt-1.4.0/doc/developers_guide/api/sbrt/shell/text/math/LinearCombFormatV1.html]

LinearCombFormatV1


|  |  |  |
| --- | --- | --- |
| **Overview** \| **Package** \| **Class** \| **Use** \| **Tree** \| **Deprecated** \| **Index** \| **Help** | |  |
| **PREV CLASS**   **NEXT CLASS** | **FRAMES**    **NO FRAMES**     **All Classes** |  |
| SUMMARY: NESTED \| FIELD \| CONSTR \| METHOD | DETAIL: FIELD \| CONSTR \| METHOD |  |


---


## sbrt.shell.text.math Class LinearCombFormatV1<V>

```
java.lang.Object
  sbrt.shell.text.math.LinearCombFormatV1<V>
```

**Type Parameters:**: `V` - the variable type.

**All Implemented Interfaces:**: Format, Formatter<LinearComb<V>>, LinearCombFormat<V>, MathExprFormat<LinearComb<V>,V>, Parser<LinearComb<V>>, SimpleFormat<LinearComb<V>>

---

``` public final class LinearCombFormatV1<V> extends java.lang.Object implements LinearCombFormat<V> ```

This class is a concrete implementation of `LinearCombFormat`. The
linear combination format defined by this class looks like:

*(coefficient_1_) variable_1_ + (constant)*

Coefficients and constants must be enclosed by parentheses. In terms, the
coefficients must appear before the variable, and the two must be separated
by at least one whitespace character. Terms must be separated by `+`
signs. Coefficients, constants, variables, and `+` signs must all be
separated from each other by at least one whitespace character, which means
variable strings cannot contain any whitespace characters. The constant in a
linear combination string can occurr in any sensible position.

**Author:**
:   This class was written and documented by Jeremiah Wright while in the
    Wagner lab.

---

**Constructor Summary**

`LinearCombFormatV1(SimpleFormat<V> variableFormat)`
  
          Constructs a new linear combination format using the provided format.

`LinearCombFormatV1(SimpleFormat<V> variableFormat,
double zeroCutoff)`
  
          Constructs a new linear combination format using the provided arguments.


| **Method Summary** | |
| --- | --- |
| `java.lang.String` | `format(LinearComb<V> expression)`             Returns a formatted string representation of the provided linear combination. |
| `static LinearCombFormatV1<java.lang.String>` | `getBasic()`             Returns a `LinearCombFormatV1` that does not format or parse the variables supplied to it. |
| `CoeffFormatV1` | `getCoeffFormat()`             Returns the format used for coefficients. |
| `LinCombTermFormatV1<V>` | `getTermFormat()`             Returns the format used for terms. |
| `SimpleFormat<V>` | `getVariableFormat()`             Returns the format used for variables. |
| `LinearComb<V>` | `parse(java.lang.String expression)`             Parses the provided string and returns its corresponding linear combination. |

**Methods inherited from class java.lang.Object**

`clone, equals, finalize, getClass, hashCode, notify, notifyAll, toString, wait, wait, wait`

**Methods inherited from interface sbrt.shell.text.Formatter**

`format`

**Constructor Detail**

### LinearCombFormatV1

```
public LinearCombFormatV1(SimpleFormat<V> variableFormat,
                          double zeroCutoff)
```

:   Constructs a new linear combination format using the provided arguments.

    **Parameters:**: `variableFormat` - the format to use for variables.: `zeroCutoff` - the zero cutoff for constants and coefficients. **See Also:**: `LinearComb.getZeroCutoff()`

---


### LinearCombFormatV1

```
public LinearCombFormatV1(SimpleFormat<V> variableFormat)
```

:   Constructs a new linear combination format using the provided format. The
    zero cutoff is set to 0.0d.

    **Parameters:**: `variableFormat` - the format to use for variables. **See Also:**: `LinearComb.getZeroCutoff()`


**Method Detail**

### getBasic

```
public static LinearCombFormatV1<java.lang.String> getBasic()
```

:   Returns a `LinearCombFormatV1` that does not
    format or parse the variables supplied to it. In other
    words, it returns a `LinearCombFormatV1` with
    the most basic possible functionality. Empty strings are not
    allowed however.

    :   **Returns:**: a `LinearCombFormatV1` that does not format or parse the variables supplied to it.

---


### getVariableFormat

```
public SimpleFormat<V> getVariableFormat()
```

:   Returns the format used for variables.

    :   **Returns:**: the format used for variables.

---


### getTermFormat

```
public LinCombTermFormatV1<V> getTermFormat()
```

:   Returns the format used for terms.

    :   **Returns:**: the format used for terms.

---


### getCoeffFormat

```
public CoeffFormatV1 getCoeffFormat()
```

:   Returns the format used for coefficients.

    :   **Returns:**: the format used for coefficients.

---


### format

```
public java.lang.String format(LinearComb<V> expression)
```

:   Returns a formatted string representation of the provided linear
    combination.

    :   **Specified by:**: `format` in interface `LinearCombFormat<V>` **Specified by:**: `format` in interface `MathExprFormat<LinearComb<V>,V>`
    :   **Parameters:**: `expression` - the linear combination to be formatted. **Returns:**: a formatted string representation of the provided linear combination.

---


### parse

```
public LinearComb<V> parse(java.lang.String expression)
```

:   Parses the provided string and returns its corresponding linear
    combination.

    :   **Specified by:**: `parse` in interface `LinearCombFormat<V>` **Specified by:**: `parse` in interface `MathExprFormat<LinearComb<V>,V>` **Specified by:**: `parse` in interface `Parser<LinearComb<V>>`
    :   **Parameters:**: `expression` - the string to be parsed. **Returns:**: the linear combination corresponding to the provided string.


---


|  |  |  |
| --- | --- | --- |
| **Overview** \| **Package** \| **Class** \| **Use** \| **Tree** \| **Deprecated** \| **Index** \| **Help** | |  |
| **PREV CLASS**   **NEXT CLASS** | **FRAMES**    **NO FRAMES**     **All Classes** |  |
| SUMMARY: NESTED \| FIELD \| CONSTR \| METHOD | DETAIL: FIELD \| CONSTR \| METHOD |  |


---
